# Supplementary material for: Multi-population Black Hole Algorithm for the problem of data clustering
Source: PLoS One. 2023 Jul 5;18(7):e0288044. doi: 10.1371/journal.pone.0288044 (PMC10321652; doi:10.1371/journal.pone.0288044)
Supplement: Attachment — Submitted filename: Response To Reviewers-m.docx [file pone.0288044.s001.docx]

| **Multi-Population Black Hole Algorithm for the Problem of Data Clustering** | |
| --- | --- |
| **Sinan Q. Salih, AbdulRahman A. Alsewari, Haneen A. Abdulwahab, Mustafa K. A. Mohammed, Tarik A. Rashid, Shadi S. Basurra** | |
| The authors would like to sincerely thank the Editor in chief and the associated editor for the time spent reviewing our manuscript and for the possibility to reconsider our revised work for publishing in this admired journal.  The authors deeply value the comprehensive comments needed to perform this revision and the valuable suggestions made by the respected reviewers.  Kindly, please find below our reply to the points raised and the corresponding modifications we have made in the revised manuscript to accommodate all the comments provided by the reviewers.  Our commitment to thoroughly revise this paper was driven by the valuable comments, and we are confident the paper has elevated to a greater level of clarity and professional contribution to the scientific outcomes.  All changes made to accommodate the referee's comments are blue-colored.  We hope the revised paper can be accepted based on overall positive comments from reviewers and our comprehensive revision of the original version. | |
| **Reviewer #1** | |
| Comment | In this work, the authors introduce MBHA a multi-population variant of the Black Holes Algorithms. MBHA was tested on 9 "easy" benchmark functions and some standard datasets. The results look promising, but further investigation is required before accepting such a work. The work is written in an acceptable English even though there are some typos and the use of special characters instead of English words (such as the &). I suggest a major revision for this work and I hope the authors will address my comments to improve the soundness in the presentation of their work. |
| Response | **We appreciate the effort you put into reviewing our manuscript thoroughly and providing us with specific feedback to enhance the clarity and overall impact of our work. Your comments have enabled us to revise and strengthen our manuscript, and we are confident that it will now make a significant contribution to the scientific literature. We are very much pleased to consider the reported concerns by your side and corrected them accordingly.** |
|  |  |
| Comment | In the abstract, the first time BHA is introduced as an acronym, there is a typo on the A. |
| Response | **We apologize for this mistake. The word Algorithm was missing. We have corrected the abstract as mentioned by the respected reviewer.** |
|  |  |
| Comment | The authors should avoid to use & as a character in the text and substitute it with the proper term: and.. |
| Response | **Thank you very much for this important comment. We have updated the manuscript by replacing (&) with (‘and’).** |
|  |  |
| Comment | In the introduction the authors provide many examples of meta-heuristics which are aslo state-of-the-arts. On the other hand they do not cite many important meta-heursitcs such as PSO, CMA-ES, the improvements of Differential Evolution. Moreover, they should be aware of Dorigo recent works that claim that Grey Wolf is just an ad-hoc case of PSO, thus increasing the need of citing the historical works that paved the way to the rising of these new metaphors and algorithms. |
| Response | **We would like to thank the reviewer for mentioning this comment. We have updated this paragraph by citing PSO, and DE algorithms.** |
|  |  |
| Comment | In the introduction the authors should make the relationship between data clustering and optimization methods more clear. In the current state, it seems (during the first reading) that are two separate worlds and they jumpy from one to another between two paragraphs. I suggest them to improve how the present the linkage between the two worlds. Otherwise, non-expert readers may be really confused and miss the point of the paper. |
| Response | **Thank you very much for your comment, we have added the following to the introduction section : “***Various real-world applications have implemented DC methods widely. The main aim of this approach is to partition data objects such that the accumulated distances between data objects and their respective centroids are minimized. By clustering, objects within a cluster should have as much similarity as possible while being significantly different from objects in other clusters. In other words, DC can be viewed as an optimization problem where the objective is to partition a given set of data points into a fixed number of clusters such that the within-cluster similarity is maximized and the between-cluster similarity is minimized*. “ |
|  |  |
| Comment | Section 2 should be named "background" or some more formal term than "preliminaries". Moreover, in the introduction of Section 2, the authors should briefly introduce the topic of each sub-section . |
| Response | **Thank you for these two comments. We have changed the title of section 2. In addition, the introduction of this section has been updated as requested by the respected reviewer.**  **“***This section aims to offer an overview of the data clustering optimization problem and the black hole optimization algorithm. First, the section explains data clustering as an optimization problem, providing the necessary mathematical formulation. Additionally, the section presents a review of the most significant related works. The second subsection explains the original version of Black Hole Algorithm (BHA), and discusses its advantages and drawbacks.***”** |
|  |  |
| Comment | The authors state that "The goal of data clustering is achieved by implementing certain similarity measures". This is not completely true: first of all, the measures typically have to be maximized or minimized, not just implemented, thus I suggest a more proper choice of words. Secondly, not always good metrics mean good clusterings, sometimes a separation that maximizes a metric might yield to clusterings that have no sense for a domain expert or clusterings that separates data according to some unmeaningful patterns. Lastly, the choice of a proper similarity measure is key. |
| Response | **Based on the reviewer's opinion, we have removed the mentioned statement.** |
|  |  |
| Comment | The authors state "Some of the common application areas of DC are image processing, analysis of medical images, as well as statistical data analysis. They are also useful in various science and engineering fields and are sometimes used interchangeably with statistical data analysis. Clustering is also used in machine learning (ML), image analysis, pattern recognition, bioinformatics, and information retrieval as the primary task for exploratory data mining. The differences across clusters can be attributed to their sizes, shapes, & densities, as seen in Figure 1." It is not clear why they are stating basically the same concept twice. |
|  |  |
| Response | **We would like to apologize for this mistake. We have removed the second statement as it states the same concepts.** |
|  |  |
| Comment | The authors state "seeking proficiency in 3 and probably three dimensions" I think that a typo here was inserted. |
| Response | **We apologize for this typo, we have removed “3 and “.** |
|  |  |
|  |  |
| Comment | The authors should explicitly state the meaning of each acronym, for instance, the ABC acronym was never introduced, also GA and PSO were never introduced. I think this is an important operation because acronyms without their explicit meaning can hinder the readings of non-expert and might prevent expert readers to disambiguate acronyms that are used to mean different algorithms/metrics/methods. |
| Response | **Thank you for mentioning this mistake for us. We have corrected these acronyms in the introduction and background sections.** |
|  |  |
| Comment | In equation (2) the authors write i=1.2. ... N, is there a reason why 1 and 2 are separated by the dots or is it just a typo? |
| Response | **This is a typo, we apologize for this. We have corrected it.** |
|  |  |
| Comment | The authors state "the BHA converges to the global optimum in all runs, unlike the other heuristics that can be trapped in locally optimal solutions". I think that this is at least a too dangerous statement. I remind the authors that the No Free Lunch Theorem also implies that a method that always converges to the global optimum in all runs (which I assume it is a synonym for problems) does not exist. Also, both the cited papers do not prove such claim "for all the runs". |
| Response | **We apologize for the misunderstanding. In BHA, any solution or star that has a fitness value less than R is removed and replaced with a new solution, thereby exploring a new solution that could be placed in a completely new position in the search space. This condition is checked in every iteration, which may decrease the chances of the algorithm getting trapped in local optima. We agree that an algorithm that always converges to the global optimum does not exist. However, in our case, BHA and other population-based algorithms perform both global and local searches.**  **Regarding the word "runs," we understand that it may have confused. We have corrected it to "iterations" to avoid any future misunderstandings. We appreciate the reviewer's comment and hope that our response adequately addresses their concern.** |
|  |  |
| Comment | This is a comment that the authors might not address in the paper, but I think it is something worth to think about: by looking at how BHA is proposed and its equation, isn't BHA a variant of PSO where the particles are only attracted towards the global optimum with the presence of a tabu region around the optimum? Moreover such tabu region does not avoid a local exploitation to find more promising optima near the black hole? |
| Response | **Thank you for your insightful comment. Indeed, we have already compared the main position updating equation of BHA to PSO and found that they are very similar. However, it is worth noting that BHA updates the positions of solutions based on the position of the best solution (BH) and the current position of the solution (*Xi*) only, while the original version of PSO involves three controlling parameters (the cognitive and social parameters *c1* and *c2*, and the inertia weight *w*). The lack of these parameters makes BHA a simpler algorithm compared to PSO.**  **We appreciate your suggestion to include this comparison in the manuscript, but we did not include it because it is not the main contribution of our work. Our focus in this paper is on enhancing BHA through a new multi-population structure.** |
| Comment | On page 20 there is a paragraph with the text with a lower font size.  Moreover, I think that this portion of sentence holds some mistakes due to editing "the search process during the early iterations is considered to be a global search () is small". |
| Response | **We apologize for this mistake; we have enlarged and checked this paragraph.** |
|  |  |
| Comment | - Line 6 of Algorithm 1 has a typo: "vie". - Line 32 of Algorithm 1, population has become a subscript of a subscript, which I don't think it was what I intended - In Figure 3 after the block of "For p=1 ..." there is a row which is not aligned with the others. - Moreover, the captions should end with a termination mark. - Also the captions of the Table should end with a termination mark. |
| Response | **Thank you for mentioning these mistakes. We have corrected all of them in the revised version.** |
|  |  |
| Comment | The tested benchmark functions in Section 4.1 is far away from being sufficient and able to provide a sound analysis. Sphere and Sumsquare are basically the same function: the global optimum is located in the same position and the only difference is the value the xs are evaluated, thus resulting in very similar landscapes. If a methods performs well on one of such two functions should perform well also in the other function. Moreover, if I made the correct math and properly searched for sources, all the benchmarks have the global optimum located in O = (0,...,0) which can be very convinient for some algorithms. As a consequence, the whole selected functions are a special case of optimization problems where the optimum might actually lie in any position of the landscape. I suggest the authors to test the algorithm on more difficult benchmark functions which can be shifted, rotated, shrinked, composed or hybridized versions of wide known benchmark functions. I suggest them to leverage some benchmark suite proposed in competitions of optimization conferences, such as CEC or GECCO and test MBHA on at least one of these suites in at least 30 dimensions. On the other hand, I appreciated the inclusion of a stochastic noisy function (i.e., Quartic). |
| Response | **The primary objective of this study is to propose a novel variant of the Black Hole Algorithm (BHA) that incorporates a new multi-population structure. Although the standard version of BHA, developed by Hatamlou in 2013, has already been shown to be a powerful optimization algorithm for data clustering problems, our focus is on using the modified version of BHA to enhance its performance in this specific application. Through our experiments, we demonstrate that the proposed variant outperforms the standard version in terms of clustering accuracy, indicating its potential as an effective optimization algorithm for data clustering tasks.** |
|  |  |
| Comment | On page 24 it is not clear why there are quotation marks around the tested methods. |
| Response | **We apologize for this mistake. We have removed these quotation marks in the revised version.** |
|  |  |
| Comment | The authors should state how and why they chose such values for the hyper-parameters of the tested algorithms. If they performed an hyper-parameter search for MBHA they should employ the same search for the other algorithms even in terms of number of iterations and populations sizes. The important part to obtain fair comparisons is the total budget in terms of fitness evaluations not to have the same number of individuals/particles and iterations for each algorithm. In addition, I suggest the authors to compare MBHA with other state-of-the-art algorithms like CMA-ES and one empowered version of Differential Evolution, such as L-Shade, jSO or even the more recent ones. |
| Response | **Thank you for your valuable comment. We appreciate your suggestion on tuning the controlling parameters of the algorithms. However, we used the default values of these parameters as they were used in the original research papers of each algorithm. We understand that tuning these parameters can potentially enhance the performance of the algorithms, but it is beyond the scope of this paper. On the other hand, the first row in Table 2 “General” presents the structural parameters which are the same for all these algorithms including BHA and MBHA.**  **Since you suggested this point, we have cited these works in the introduction, We will consider your suggestion for future modifications. Thank you again for your input.** |
| Comment | Evaluating the mean and standard deviation of the results is not enough, sound statistical tests must be performed. Ranking tests with post-hocs correction should be the proper choice to assess the most performing algorithms among the tested ones. |
| Response | **Indeed, this is an important comment. We have added another comparison between MBHA and other algorithms based on Wilcoxon Signed Rank test. Table 4 presents this comparison. Thank you for this comment.** |
|  |  |
| Comment | Figures from 4 to 9 can be collapsed together in a unique figure thus saving space and easing the reading process. |
| Response | **We agreed with the reviewer on this comment. We have removed the 3D plotting of the functions, and kept the convergence figures only. In the updated version, these figures are more readable than the original version of the manuscript. The figures are collapsed as suggested by the reviewer.** |
|  |  |
| Comment | In page 30 there is a capital letter after the ':' sign. |
| Response | **Thank you for this comment. It has been corrected.** |
|  |  |
| Comment | In Section 4.2 is not clearly stated what is the objective function the algorithms are optimizing and how it is computer. Moreover, I think that a more solid choice of the dataset should be made. I understand that labeled datasets are needed to evaluate the performance, on the other hand the authors are using a clustering approach simply as a classification task, which cmight not always be the case. Thus I suggest them to at least consider some benchmark datasets that were proposed for clustering tasks. |
| Response | **Thank you for these important comments.**   - **The objective function has been mentioned in section 2.1, equation 1. Which represents the sum of intra clusters distances. The goal is to minimize these distances.** - **We appreciate the reviewer's comment regarding the use of the datasets. We agree that testing on various datasets would provide a more comprehensive evaluation of our proposed algorithm. However, we chose to use the same datasets as in the original version of the black hole algorithm and other related works to ensure a fair comparison of MBHA with existing methods. Utilizing different datasets would make it difficult to compare the performance of MBHA with other algorithms that also used these datasets. Therefore, we decided to keep the dataset selection consistent with previous works. Nonetheless, we appreciate the suggestion and acknowledge the importance of testing on multiple datasets. The set of datasets has been used for the evaluation process previously by many researchers, such as :** - **10.1016/j.ins.2012.08.023** - **10.1016/j.eswa.2011.05.027** - [**10.1016/j.asoc.2016.04.026**](https://doi.org/10.1016/j.asoc.2016.04.026) - **10.1142/S0218213015500335** - **10.1016/j.knosys.2018.09.013** |
|  |  |
| Comment | The conclusion and the future works are poorly presented. |
| Response | **Thank you for this comment. The conclusion section has been rewritten accordingly.** |

| **Reviewer #2** | |
| --- | --- |
|  |  |
| Comment | The paper is interesting and mostly well written. I have the following comments: |
| Response | **We do highly appreciate your valuable general point of view. Your comments have been reflected accordingly.** |
|  |  |
| Comment | - Change "The second section" to Section 2  - Change "Finally, section 5" to "Finally, Section 5"  - Change "data set" to "dataset"  - The adverb "where" after any equation should be written with small letters. |
| Response | **Thank you for these comments. The requested modifications have been reflected and highlighted for your reference for tracking.** |
|  |  |
| Comment | The proposed approach should be compared experimentally or theoretically to well-known multi-population algorithms such as  An improved harmony search algorithm for solving optimization problems  Island-based Cuckoo Search with Highly Disruptive Polynomial Mutation  Distributed Grey Wolf Optimizer for scheduling of workflow applications in cloud environments |
| Response | **We agree with the reviewer’s point of view. We have reviewed the mentioned papers by the respected reviewer in the introduction section.** |
|  |  |
| Comment | - The limitations of the study should be mentioned in the conclusion section of the paper  - The authors wrote in the conclusion " In the future, more benchmark problems must be used to verify the MBHA algorithm. ". You should mention examples of the benchmark problems in this sentence. |
| Response | **Thank you for these excellent comments. We have revised the conclusion accordingly.** |
|  |  |

| **Reviewer #3** | |
| --- | --- |
| Comment | A modified version of the recent metaheuristic Black Hole Algorithm is applied for clustering. The experiments are well organized and thorough.  The text necessitates revisions, the authors should re-read the entire manuscript to get rid of the small issues that appear here and there. |
| Response | **Thank you very much for taking the time to review our manuscript. Your insightful comments and constructive criticism have been invaluable in improving the quality of our research. We have updated our manuscript based on your important comments.** |
|  |  |
| Comment | There are a few more recent metaheuristics that are validated on function optimization as in this study and then used within a ML approach and they should be referred in the section where the state of the art is presented:  https://doi.org/10.3390/math9161929  https://doi.org/10.1007/978-981-16-3728-5_1  https://link-springer-com.am.e-nformation.ro/chapter/10.1007/978-3-030-37826-4_6  https://doi.org/10.3390/math10224173  <https://doi.org/10.1016/B978-0-323-85117-6.00005-4> |
| Response | **Thank you for this comment. Indeed, these are important references. We have updated the manuscript based on the suggestion of the respected reviewer.** |
|  |  |
| Comment | The introduction of clustering in the abstract is too basic, it looks like it is addressed to readers from a domain different from computer science. The following statement from the abstract is not clear: “The original BHA’s performance was better when implemented on a benchmark dataset, even though its exploration capability is poor.” Neither the first sentence, nor the second make sense. Actually, the second statement is repeated as well in the article and it is not justified there either. This happens in the first sentence of section 3, “The standard version of BHA does not have exploration capabilities”. Please either provide a citation, if this was demonstrated anywhere before or, if this is demonstrated in the current study, this should be mentioned, because this affirmation does not represent an obvious certitude. |
| Response | **The authors very much apologize for this confusion.**   - **We have revised the abstract as follows :**   *“The original BHA algorithm showed better performance compared to other algorithms when applied to a benchmark dataset, despite its poor exploration capability.”*   - **We have revised and cited the first statement in section 3 as follows:**   *“The weakness in the exploration capability of the Black Hole Algorithm (BHA) stems from its low diversity population. The algorithm tends to converge too quickly to local optima, which limits its ability to explore the search space and find global optima.”* |
|  |  |
| Comment | In the introduction, it is stated that “newly, a multi-swarm or a multi-population has been incorporated into a series of metaheuristics such as …”. Multi-population is not a novel approach, it has been used for many years especially for dealing with multi-model problems: https://ieeexplore.ieee.org/document/1501611,  <https://doi.org/10.1007/978-3-642-01885-5_3> |
| Response | **We appreciate and fully acknowledge the reviewer's comment. We intended to highlight the established and effective use of the multi-population approach in optimization algorithms for over a decade, as the reviewer has pointed out. We have revised the statement accordingly and provided appropriate references to support this point. Thank you for bringing this to our attention.** |
|  |  |
| Comment | Figure 1 should be better explained in the caption. |
| Response | - **Thank you for this comment. We have updated Figure 1 accordingly as follows:** - **“***Figures 1. (I) and (II) the difference between the data before and after performing the clustering*”**.** |
|  |  |
| Comment | Just before subsection 4.1, there is an unfinished statement “Secondly, the application of data clustering.” |
| Response | **We apologize for this mistake. We have updated this paragraph as follows :**  **“***Secondly, the MBHA algorithm has been validated and tested based on six benchmark datasets, and compared to other powerful state-of-art algorithms*.” |
|  |  |
| Comment | The title of subsection 4.2 should be revised. Maybe “based” could be removed, please check. |
| Response | **We have removed the word “based” as suggested by the respected reviewer from subsection 4.2.** |
