# Supplementary material for: Multi-population Black Hole Algorithm for the problem of data clustering
Source: PLoS One. 2023 Jul 5;18(7):e0288044. doi: 10.1371/journal.pone.0288044 (PMC10321652; doi:10.1371/journal.pone.0288044)
Supplement: Attachment — Submitted filename: Response To Reviewers-18-5.docx [file pone.0288044.s002.docx]

| **Multi-Population Black Hole Algorithm for the Problem of Data Clustering** | |
| --- | --- |
| **Sinan Q. Salih, AbdulRahman A. Alsewari, Haneen A. Abdulwahab, Mustafa K. A. Mohammed, Tarik A. Rashid, Debashish Das, Shadi S. Basurra** | |
| The authors would like to sincerely thank the Editor in chief and the associated editor for the time spent reviewing our manuscript and for the possibility to reconsider our revised work for publishing in this admired journal.  The authors deeply value the comprehensive comments needed to perform this revision and the valuable suggestions made by the respected reviewers.  Kindly, please find below our reply to the points raised and the corresponding modifications we have made in the revised manuscript to accommodate all the comments provided by the reviewers.  Our commitment to thoroughly revise this paper was driven by the valuable comments, and we are confident the paper has elevated to a greater level of clarity and professional contribution to the scientific outcomes.  All changes made to accommodate the referee's comments are blue-colored.  We hope the revised paper can be accepted based on overall positive comments from reviewers and our comprehensive revision of the original version. | |
| **Reviewer #1** | |
| Comment | I would like to thank the authors for their kindness and answering to my comments. Unfortunately, before endorsing the publication of the manuscript I point out some minor changes that need to be addressed. |
| Response | **We are very much pleased to consider the reported concerns by your side and corrected them accordingly.** |
|  |  |
| Comment | The acronym of the proposed algorithm introduced in the abstract is BHÀ, while for the rest of the paper BHA is used. The authors should fix this typo. |
| Response | **We apologize for this mistake. The acronym has been corrected.** |
|  |  |
| Comment | Regarding the settings of the tested optimization algorithms in Section 4.1, I suggest them to explicitly state that the settings used for the compared algorithms are the values used in the original papers. |
| Response | **Thank you very much for this important suggestion. We have updated the manuscript by adding the following to section 4.1**  **“***These parameter settings were utilized in their default values as specified in the original versions.***“** |
|  |  |
| Comment | For the comment regarding the choice of the dataset in Section 4.2, I thank the authors for their answer and I kinda agree with them. However, I suggest them to explicitly state in the paper that the datasets were chosen to perform a fair comparison according to what was done in previous works. |
| Response | **Thank you very much for this important suggestion. We have updated the manuscript by adding the following to section 4.2**  **“***To ensure a fair comparison with existing methods, the same datasets used in the original version of the black hole algorithm and related works were utilized. Using different datasets would make it difficult to compare performance. Although testing on multiple datasets is important, consistency in dataset selection was prioritized.****”*** |
| Comment | In the conclusion Section, I would remove the first term "Presently". |
| Response | **The word ‘Presently’ has been removed from the conclusion section as suggested by the respected reviewer.** |
|  |  |
| Comment | In the "Support Vector Machine(SVM)" a whitespace is lacking at the end of "Machine". |
| Response | **We apologize for this mistake. We have corrected it in the revised version.** |
